# The impact of room shape on affective states, heartrate, and creative output

**DOI:** 10.1016/j.heliyon.2024.e28340

**Published:** 2024-03-16

**Authors:** K. Strachan-Regan, O. Baumann

**Affiliations:** Bond University, School of Psychology, Robina, Australia

**Keywords:** Built environment, Neuroarchitecture, Environmental psychology, Emotion, Creativity

## Abstract

The architectural design of space can deeply impact an individuals' mood, physiology, and mental health. While previous research has predominantly focused on elements like nature and lighting within architectural spaces, there is a growing literature base that also investigates the psychological and neurophysiological impacts of geometrical properties of architectural spaces. Employing virtual reality technology, the study sought to investigate the effects of curved and rectangular architectural spaces on affective states, heart rate, and creativity.

A total of 35 participants were exposed to two distinct virtual environments: a curved room and a rectangular room. Participants' self-reported mood was assessed using the Positive and Negative Affect Schedule (PANAS-Long Form). Heart rate was monitored using a pulse oximeter, and creative output was evaluated using the Guilford Alternative Uses Task (GAUT).

Statistical comparisons between the two room types indicated that participants experienced higher positive affect and lower negative affect in the curved room condition compared to the rectangular room condition. Furthermore, heart rate measurements revealed lower physiological arousal in the curved room. Additionally, participants exhibited higher creative output in the curved room as opposed to the rectangular room.

These findings align with previous literature on the influence of geometric factors on affective responses. The implications of this study are significant as they pertain to individuals' daily environments and their impact on health and well-being. The positive influence of curved room geometry on mood, arousal, and creativity emphasises the importance of considering room layout and design in various settings, such as workplaces and educational environments. Architects and designers can utilise these findings to inform their decisions and promote neuroarchitecture that enhances positive emotional experiences and productivity.

## 1 Introduction

Architectural design significantly influences the mood, physiology, and mental health of individuals, extending its impact from personal spaces to workplaces and rehabilitation centres [ [[1], [2], [3]]]. As the vast majority of individuals predominantly inhabit indoor environments [4] it becomes imperative to understand the impact of architecture on individuals' overall well-being. Previous studies have shown that architectural features such as room size, room colour, and window size positively impact human emotional responses and reduce stress [[5], [6], [7], [8]].

While previous research has predominantly focused on elements like nature and lighting within architectural spaces, there is a growing literature base that also investigates the psychological and neurophysiological impacts of geometrical properties of architectural spaces, indicating an emotional preference for room curvature and complex differences in brain activity to different degrees of room curvature [[9], [10], [11]]. Preference for curvature, both in 3D and 2D space, has been extensively investigated. Across studies, there's a consistent affinity for curved shapes and objects, which are perceived as more pleasant and beautiful than angular ones [12]. This preference extends beyond abstract shapes, as evidenced by the higher aesthetic preference for curved designs in various domains such as typography, car interiors, and common objects [13,14]. The preference for curvature appears to be innate and may develop early in life, as observed in infants and young children [15,16]. Cross-cultural studies suggest that the preference for curvature is universal rather than culture-specific [17].

Interestingly, research suggests that several features of rooms, such as complexity of visual detail, use of natural materials, warmer colours, and views of nature, have a positive effect on creativity [18,19]. This suggests a potential impact of room shape on creativity and its potential to conduce creative thinking, which is relevant to workplace or educational settings.

Limited research has observed the association between curved and rectangular environments and their effects on creativity. The current study therefore aims to investigate individuals' creative and affective responses to curved and rectangular architectural spaces. A converging procedures approach, including subjective self-reported affect, as well as objective measures like heart rate and a standardised creativity task, was employed to assess the impact of these environments.

Finally, real-world spaces pose limitations due to uncontrollable environmental factors. Virtual reality (VR) technology offers an alternative approach, providing controlled and immersive environments for research purposes [20]. VR has been previously used successfully to evaluate subjective mood (self-rated valence mood and arousal) and objective mood (physiological arousal), while being able to create a realistic reproduction of built environments that control for architectural features as well as participants viewing perspective [7,20,21]. The current study therefore aimed to investigate individuals' creative and affective responses to curved and rectangular architectural spaces using virtual reality.

H1 Participants' self-reported affective response would be significantly influenced by room geometry. Specifically, it was predicted that subjective positive affect would be significantly higher in the simulated curved-room condition compared to the rectangular-room condition, while keeping room size (floor area and height) and window size constant (H1a). Additionally, it was predicted that the rectangular-room condition would elicit a significantly higher negative affect compared to the curved-room condition (H1b).

H2 Participants' heart rate, measured in average heartbeats per minute, would be significantly lower in the simulated curved-room condition compared to the simulated rectangular-room condition, indicating lower arousal.

H3 Participants creative output, measured by The Guilford's Alternate Uses Task (GAUT) would be higher in the curved-room condition than in the rectangular-room condition.

## 2 Materials and methods

### 2.1 Design

**Fig. 1:**
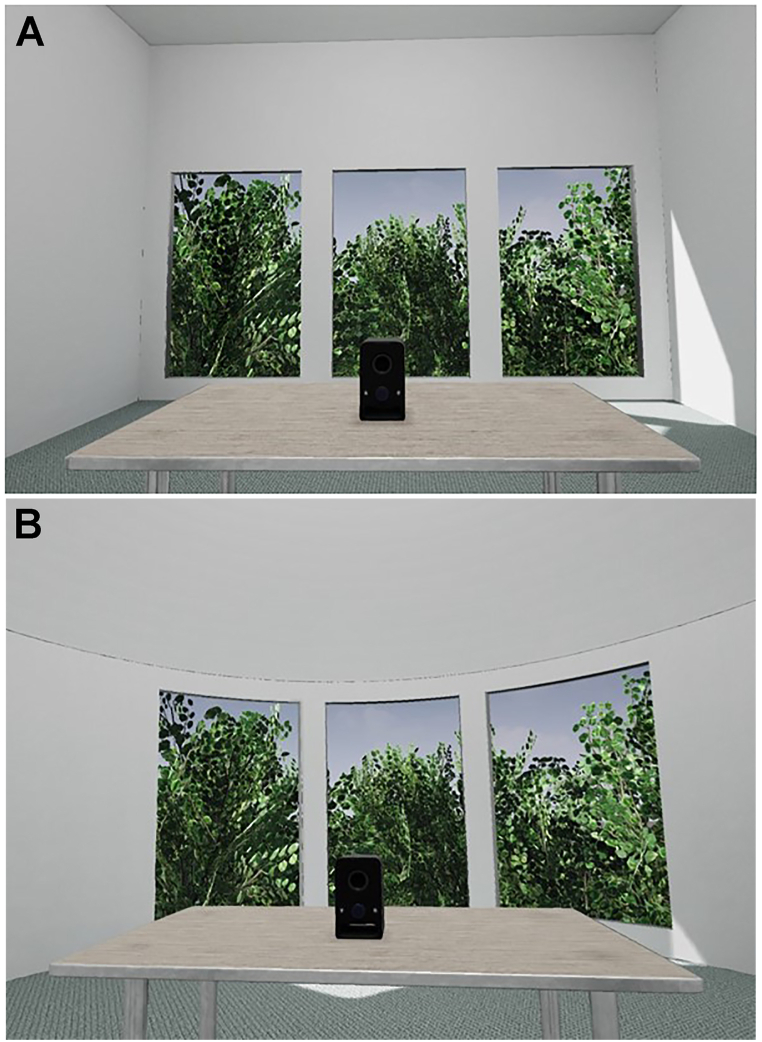
The Simulated (A) Rectangular Room Environment and (B) Curved Room Environment. *Note.* Both rooms were white, and approximately 20 m^2^ in floor area, and 2.8 m ceiling height. A) The *rectangular* room had a square window and dimensions. B) The *curved* room featured rounded walls and windows and dome-shaped ceiling.

The study employed a within-subjects, counterbalanced AB/BA design with two virtual reality scenes: the rectangular room (see Fig. 1A) and the curved room (see Fig. 1B). The order of the environments was counterbalanced across participants. The study had one independent variable (room shape) with two conditions: rectangular room and curved room. The dependent variables included affect (PANAS-Long Form), Guildford Alternative Uses Task (GAUT), and heart rate.

### 2.2 Participants

The current study utilised a sample of 35 participants, with ages ranging from 18 years to 64 years (M = 25.6, SD = 9.11). Participants were recruited through the University's Research Participant Pool and social media platforms. Inclusion criteria required participants to be proficient in English and have near normal or corrected vision; all participants satisfied these requirements. The sample consisted of 15 males (42.9%) and 20 females (57.1%), with 18 participants (51.4%) being students, 13 (40%) employed, and 3 (8.7%) unemployed. Ethics approval was acquired via the Bond University Human Research Ethics Committee (Ethics approval number: #OB00022) and informed consent was obtained from all participants prior to the start of the experiment.

## 3 Materials

### 3.1 PANAS

The Positive and Negative Affect Schedule (PANAS-LF) was used, which is a self-report questionnaire designed to measure an individual's affect and current emotional state [35]. The PANAS assesses positive affect and negative affect as distinct mood dimensions. Participants are presented with words denoting specific emotional states, which they then rate based on their immediate feelings using a five-point Likert scale, ranging from 1 (very slightly or not at all) to 5 (extremely). Positive affect words include “excited,” “inspired,” and “enthusiastic,” while negative affect words include “irritable,” “afraid,” and “nervous.” Total raw scores are calculated for positive affect and negative affect separately, ranging from 10 to 50, with higher scores indicating higher levels of the corresponding mood dimension. The PANAS has demonstrated good internal consistency, with an observed Cronbach's alpha of α = 0.89 for positive affect and α = 0.85 for negative affect as well as high convergent validity compared to other validated affect scales [22,23].

### 3.2 Heart rate measurement

A heart rate monitor, specifically the Elite HRV CorSense device, was used to measure participants' average heart rate in beats per minute. The Elite HRV mobile application was used to record and analyse the heart rate data. The device was attached to participants' index fingers with their consent. Previous studies have shown that negative emotions can be associated with higher heart rate, while lower resting heart rate is linked to positive mood [24,25], and heartrate has been used in previous VR studies to measure physiological arousal [20,26]. The Elite HRV software includes signal quality algorithms to detect false heartbeats and ensure accuracy. The CorSense device and software has been successfully used in various studies measuring heart rate [[26], [27], [28], [29]].

### 3.3 Creativity measurement

#### 3.3.1 Guildford alternative use task

The Guilford's Alternate Uses Task (GAUT) is a well-established instrument for assessing divergent thinking, a component of creativity [30,31]. To assess divergent thinking, participants are prompted to generate multiple unconventional uses for commonplace items, within a specified time frame [32]. The GAUT task encourages participants to diverge from traditional frameworks of thought (e.g., a knife could be used for cutting), to include novel perspectives and creative reinterpretations of objects (e.g., a knife could be used as a mirror). Prior research utilising the GAUT, incorporated objects such as brick, newspaper, or knife [33]. These items underwent separate testing in a pilot study with five participants. Results from this preliminary analysis indicated that both “newspaper” and “knife” elicited higher creative responses within the GAUT, hence their selection for the primary investigation.

#### 3.3.2 Creativity assessment

To assess divergent thinking, the GAUT task traditionally relies on the subjective judgement of human raters to determine whether individual responses are both novel and creative [32] Acknowledging the potential limitations introduced by subjective scoring, the computational Semantic Distance (SemDis) platform, designed by Beaty and Johnson [34] was employed to quantitatively calculate the semantic relatedness of the GAUT responses. This approach aligns with the associative theory of creativity, which posits that creative thinking involves linking remote concepts in semantic memory (i.e., establishing connections between ideas or words that are typically unrelated; [35]. For instance, the words “hammer” and “tissue” would typically have a higher semantic distance and lower similarity score, given their dissimilar contexts. Conversely, the words “hammer’ and “nail” are more likely to occur in similar contexts, yielding a lower semantic distance score. This theory further implies that individuals with a high degree of creativity may demonstrate a more flexible semantic network structure, which could facilitate divergent thinking [35].

The SemDis platform consists of five corpus-based semantic models that are publicly available. These models were assessed by Beaty and Johnson [34] using various measures and creativity tasks. They have demonstrated their ability to predict human creativity and novelty ratings reliably across different creativity tasks, including the GAUT. For our study, we selected the CBOW model by Baroni, which was identified as a reliable predictor of creativity in Beaty and Johnson's assessment. This CBOW model was constructed by Baroni et al. [36] through a large-scale evaluation using the continuous bag-of-words (CBOW) variant of the Word2Vec algorithm. The CBOW model is a machine learning tool and neural network model designed to learn word associations from a large corpus of text. To build the CBOW model, Baroni and colleagues [36] applied the CBOW variant of Word2Vec to a corpus comprising approximately two billion words. This corpus consisted of an English Wikipedia dump, the British Web (ukWaC) corpus, and the British National Corpus. Each word in the corpus's vocabulary is mapped to a vector in a multi-dimensional space, with the position of each vector determined by its numeric values. These vectors capture the semantic relationships between words as reflected in the patterns of usage within the training dataset [34]. The CBOW model is trained to predict a target word based on its contextual usage, aiming to find words that are used interchangeably or have similar meanings within a sentence. This is represented by vectors that are close together in the vector space. The model assigns semantic scores to indicate the distance between words, ranging from −1 to 1. Higher scores suggest greater distance, implying that words with higher scores are more distantly related ideas or concepts [34]. Among the compositional models available in SemDis, multiplicative model was used, which has shown stronger alignment with human creativity ratings according to Beaty and Johnson [34] and Mitchell and Lapata [37].

Prior research has emphasised the necessity of data cleaning to improve the accuracy of CBOW models, particularly when handling phrases or multi-word responses [34]. As many GAUT responses from our study contained phrases, it was imperative to conduct thorough data cleaning prior to SemDis use, as the presence of these phrases, lack of specificity and “stop words” (e.g., an, the, but) can confound the semantic distance score or complicate the task of mapping word vectors [34]. For the current study, the following rules were implemented to clean and prepare the data prior to SemDis use.

1) The elimination of unclear or insufficiently specific responses. If GAUT responses lacked specificity or were unclear, they were removed to ensure that data was specific. This is a crucial aspect of data preprocessing, known as data cleaning or scrubbing, which maintains that the specificity of data is vital for a model, such as CBOW, to form accurate and consistent semantic associations [38].
2) The removal of responses which fail to detail a distinct application of the object. GAUT responses which did not capture a specific use for the object or focused on the object itself rather than a possible use (i.e., “throw,” “deliver,” “recycle,” or “sharpen”) were removed. This is in alignment with Han et al. [39], research, which emphasises the importance of ensuring data relevancy prior to semantic data modelling or analysis. Further, this approach is known to enhance the pertinence of the data.
3) The selection of appropriate one-word descriptors. To avoid semantic distance scores being skewed by “stop” words or phrases, we assigned a singular, pertinent descriptor to each object's use. For instance, the phrase “open lock” was simplified to “open”, while “make funnel” was represented by “funnel”. This step resonates with the text simplification principle, a common practice in data preprocessing for natural language tasks (NLP), which aim to improve the model's performance by reducing data complexity [40].
4) Conversion of verbs to their infinitive form. To reduce linguistic variation within our data, all verbs within GAUT responses were converted to their infinitive form, minus the “to,” where appropriate (e.g., “slashing tyre” wouldn't become, “to slash,” but instead “slash”). This approach, which is a form of lemmatisation, is a common natural language processing task aimed to reduce complexity of data and facilitate more accurate semantic mapping [40].
5) The elimination of duplicate responses. Any duplicate responses from the GAUT dataset were eliminated. This step aimed to remove redundancies which could skew semantic distance scores [38]. For instance, if a participant had listed both “cutting food” and “cutting string, " responses would become “cutting” and count as one response.
6) Retention of typical uses. Typical uses for objects were not removed from the GAUT dataset, as the SemDis model is able to distinguish between conventional and novel associations, assigning lower scores to typical uses due to their higher prevalence in language use and proximity in the semantic space [34]. For instance, a typical use for a newspaper may be “reading the classifieds,” which would be simplified to “reading,” while an unconventional use “making a funnel,” would be represented by “funnel.” This approach ensured that all dimensions of participants' divergent thinking were considered in the analysis, including conventional connections [34].

Finally, the software-generated Semantic Distance (SemDis) scores were summed across all the answers provided by each participant in each condition. This comprehensive score takes into account both the quantitative and qualitative aspects of participants' creativity within each condition.

### 3.4 Virtual reality environment

The virtual reality (VR) environments were simulated using Unreal Engine™ version 4 and SteamVR™ version 1.8.6 software running on a DELL Precision 5820 computer with an Intel Xeon 4.Ghz CPU, 32 GB RAM, and NVIDIA GeForce GTX1080 Graphics Card. Tracking devices were used to monitor participants' head position and orientation in space while wearing the VIVE™ VR headset, which had a resolution of 2160 x 1200 pixels.

Two VR environments were designed to simulate the rectangular room and curved room conditions (see Fig. 1). Both rooms featured office-like elements such as a wooden desk, black speaker, carpet, one door, and one light. The walls of both rooms were white, and approximately 20 m^2^ in floor area, and 2.8 m ceiling height. The main difference between the conditions was the window shape and room shape. The rectangular room had a square window and rectangular dimensions and corners, while the curved room had a curved window and curved room dimensions, including a dome shaped ceiling. Both conditions provided a view of a blue sky, trees, and greenery. To allow for replications of our study we made the virtual environment available on the Open Science Framework (please see Data Availability Statement below).

### 3.5 Procedure

Upon arrival, the informed consent and information sheet was provided. This contained information pertaining to study, ethics approval and components involved. The participants were informed to notify the researcher, if at any time, they felt uncomfortable. Once wearing the VR headset, approximately 3 min were allowed for participants to familiarise themselves with the environment. According to Yin et al. [41] this period is adequate for psychological adjustment and environmental immersion. During this time, the Elite HRV software recorded participants' heart rate activity. Subsequently, PANAS and GAUT were administered through a speaker device with participants responding verbally. Alongside VR conditions, GAUT items were counterbalanced between “knife” and “newspaper” allowing participants 60 s to list as many alternative uses which they could think of. Participants were exposed to each of the virtual environments for 10 min, which was deemed sufficient in eliciting cognitive and emotional effects [42]. Upon completion of the first condition, heart rate activity stopped recording for the first environment. This process was then repeated for the other room condition. Participants were asked to keep their eyes closed during the period between conditions while the environment was changed. The entire experiment took approximately 26 min, and participants wore the headset the entire time. Motion sickness was avoided by having participants seated and asking them not to look around during the experiment.

## 4 Results

**Fig. 2:**
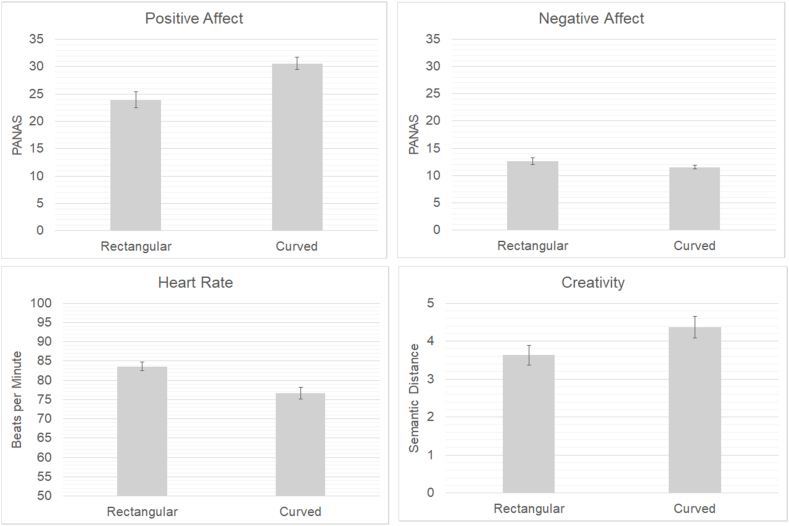
Average alues For Self-reported Mood, Heart Rate and Creativity. Note. Error bars = Standard Error (SE).

Statistical analyses were conducted using IBM SPSS-27 software, and an alpha level of 0.05 was used to assess statistical significance (see Fig. 2 for descriptive results).

A power analysis was conducted using G*Power 3.1 to determine the minimum sample size required for adequate power [43]. The analysis indicated that a paired samples *t*-test with 34 participants would be sensitive to Cohen's d = 0.50 with 80% power (alpha = 0.05, two-tailed). The final sample consisted of 35 participants, ensuring sufficient power for subsequent analyses.

### 4.1 Data screening

The HRV Elite heart rate monitor indicated poor connectivity for one participant, and their data was omitted from heart rate analysis (n = 34).

### 4.2 Data analysis

The assumption of normality was assessed via Shapiro-Wilk tests applied to the difference scores of the dependent measures. The assumption of normality was met for all dependent variables with the exception of negative affect and heart rate (Shapiro-Wilk test, *p <* 0.001). While paired sample *t*-test analyses are robust to moderate departure from normality, it is not preferable to rely on this feature [44]). Thus, to allow for greater comparability and consistency, a conservative non-parametric Wilcoxon test was conducted to compare the robustness of the paired sample *t*-test analyses for the negative affect measure and heart rate.

Positive and Negative Affect: Two paired sample t-tests (n = 35) were conducted to assess differences in positive affect scores between the curved room and rectangular room. Participants reported significantly higher scores on positive affect for the curved-room condition (M = 30.60, SD = 6.72) compared to the rectangular-room condition (M = 23.94, SD = 8.69, t(34) = 4.87, p < 0.001), with a large effect size (Cohen's d = 0.82). Additionally, participants had significantly higher negative affect scores for the rectangular-room condition (M = 12.62, SD = 3.80) compared to the curved-room condition (M = 11.51, SD = 1.99, t(34) = 2.09, p = .04), with a small effect size (Cohen's d = 0.35). Importantly, the comparative non-parametric Wilcoxon test still indicated a significant effect (Z = 1.97, p = 0.049), indicating the outcomes of the paired *t*-test were robust.

Heart Rate: A paired sample *t*-test (n = 34) was conducted to assess differences in heart rate mean scores between the curved room and rectangular room. Participants had a significantly lower heart rate for the curved-room condition (M = 76.67, SD = 8.76) compared to the rectangular-room condition (M = 83.58, SD = 6.68, t(33) = 4.18, p < 0.001), with a medium effect size (Cohen's d = 0.71). Also here, the comparative non-parametric Wilcoxon test still indicated a significant effect (Z = 3.52, p < 0.001), indicating the outcomes of the paired *t*-test were robust.

Creativity: A paired sample *t*-test (n = 35) was conducted to assess differences in Semantic Distance (SemDis) scores between the curved room and rectangular room. Participants had a significantly higher SemDis scores for the curved-room condition (M = 4.37, SD = 1.68) compared to the rectangular-room condition (M = 3.63, SD = 1.55), t(34) = 2.06, p < 0.047), with a small effect size (Cohen's d = 0.35).

## 5 Discussion

The current study aimed to investigate the impact of curved and rectangular geometrical criteria of architectural space on affect, creativity, heart rate. The hypotheses proposed in the study were all supported by the results, which will be discussed in this section.

### 5.1 Positive and Negative Affect

The study hypothesised that participants would experience higher positive affect in the curved-room condition compared to the rectangular room condition (H1a). Conversely, it was hypothesised that participants would experience higher negative affect in the rectangular-room condition compared to the curved-room condition (H1b). The findings of the study supported H1a, indicating that participants reported significantly higher positive affect scores in the curved-room condition compared to the rectangular-room condition. Additionally, H1b was also supported, as participants reported significantly higher negative affect scores in the rectangular-room condition compared to the curved-room condition.

The results align with previous literature suggesting that geometric factors, such as curvature and abstract novel shapes, can influence individuals' affective responses. Curved architectural spaces have been associated with feelings of safety, pleasantness, and reduced threat, leading to higher positive emotional responses [14,[45], [46], [47]]. In contrast, angular geometry and rectangular interior spaces can elicit a more threatening response, consistent with higher negative affect [14,15]. These findings support the notion that humans have an inherent inclination to approach environments that evoke positive emotions and avoid those perceived as unfriendly or threatening. A potential rational for this curvature preference could be its ubiquity in natural settings. Significantly, the present study extends previous research by investigating these relationships in 3D peripersonal space, using simulated built environments.

### 5.2 Heart rate

The study hypothesised that participants' heart rate would be lower in the curved-room condition compared to the rectangular-room condition (H2). The results supported this hypothesis, indicating that, on average, participants had a lower heart rate in the curved room compared to the rectangular room, suggesting lower arousal. Lower resting heart rate is associated with subjective feelings of relaxation and comfort, as well as positive affect [[48], [49]]. Prior research using functional magnetic resonance imaging and EEG have also identified corroborating findings in response to curved room conditions, indicating a neurophysiological positive emotional response [11]. While previous studies have not specifically focused on heart rate in relation to room geometry, the current study's results are consistent with the suggestion that heart rate can be a suitable way to detect physiological and subjective emotional states related to room geometry.

### 5.3 Creativity

The study hypothesised that participants' creativity would be higher in the curved room condition compared to the rectangular-room condition (H3). The results supported this hypothesis, indicating that, on average, participants had a higher creative output in the curved room compared to the rectangular room. While previous research has not specifically focused on room shape as an influencing factor for creativity, it has identified various room features as potential enhancers of creative thinking. Elements such as window views of nature, complexity, use of natural materials, warmer colours, have a positive effect on creativity [18,19]. Taken together, these findings highlight the important influence that the design and layout of ones’ space can enhance creative thinking processes.

### 5.4 Strengths, limitations, and future recommendations

A significant strength of the study is the incorporation of both objective and subjective measures. By combining these measures, the study provides a comprehensive understanding of the relationship between room geometry and affect. Additionally, using VR to investigate and display curved and rectangular room environments allowed for experimental control and replication of past results, making VR an immersive and suitable tool for studying the impact of stimuli on human emotional states.

However, the study also has limitations. Firstly, while the GAUT and semantic distance are one way to assess creativity, there are other instruments available to could be equally or more sensitive measures (see El-Murad and West [50] for an overview). By concentrating on rectangular versus curved geometry, we aimed to keep the experiment brief and prevent participant fatigue. However, future studies should explore various room shapes and curved walls [5,6]. Similarly, the potential moderating impact of other factors, such as personality of the participants are interesting variables that require further research.

## 6 Conclusions

The findings of the study have significant implications, as they relate to the settings that most humans are exposed to daily and their potential impact on individuals. The study demonstrated that rectangular room geometry can elicit increased negative mood, while curved room geometry can enhance positive mood and result in lower arousal. These affective and physiological outcomes are relevant, as they directly relate to psychological and physical health outcomes. Moreover, the positive impact of curved room geometry on creativity highlights the importance of considering room layout and design for educational and work environments.

The theoretical significance of this study lies in its contribution to filling a critical gap in the existing literature. By systematically investigating the effects of room shape on creativity and mood within a simulated environment, this research provides empirical evidence that substantiates and extends our understanding of the psychological impact of architectural design. More specifically, the findings highlight the potential positive consequences that curved spaces can elicit in terms of mood, physiological arousal, and creativity. From a practical perspective, architects and designers can use these findings to maximise positive impacts of environments on health and mental well-being. By incorporating curved room designs, they can create spaces that improve our mood, reduce stress, and increase creative thinking. In summary, our findings support the notion that room shape can significantly impact individuals' emotional experiences and creative productivity, emphasising the importance of considering this factor in architectural design. Future studies will be needed to investigate the effects on creativity of additional potentially relevant architectural elements, such as other geometric shapes, room size, and texture.

## Ethics approval

All procedures performed in this study were approved by Bond University Human Research Ethics Committee (Ethics approval number: #OB00022) and are accordance with the ethical standards as laid down in the 1964 Declaration of Helsinki and its later amendments.

## Additional information

No additional information is available for this paper.

## Data Availability Statement

The virtual environments and data associated with this study has been deposited at Open Science Framework (https://osf.io/mryus/?view_only=62f165df2eb74276a96d68ff80cba016)

## CRediT authorship contribution statement

**K. Strachan-Regan:** Writing – review & editing, Writing – original draft, Methodology, Formal analysis, Conceptualization. **O. Baumann:** Writing – review & editing, Writing – original draft, Resources, Methodology, Formal analysis, Conceptualization.

## Declaration of competing interest

The authors declare that they have no known competing financial interests or personal relationships that could have appeared to influence the work reported in this paper.
